# Expression of insulin-like growth factor-II mRNA-binding protein-3 as a marker for predicting clinical outcome in patients with esophageal squamous cell carcinoma

**DOI:** 10.3892/ol.2014.2465

**Published:** 2014-08-20

**Authors:** AKIHIRO TAKATA, SHUJI TAKIGUCHI, KAORU OKADA, TSUYOSHI TAKAHASHI, YUKINORI KUROKAWA, MAKOTO YAMASAKI, HIROSHI MIYATA, KIYOKAZU NAKAJIMA, MASAKI MORI, YUICHIRO DOKI

**Affiliations:** 1 Department of Gastroenterological Surgery, Graduate School of Medicine, Osaka University, Suita, Osaka 565-0871, Japan; 2 Department of Surgery, Nishinomiya Municipal Central Hospital, Nishinomiya, Hyogo 663-8014, Japan

**Keywords:** insulin-like growth factor-II mRNA-binding protein-3, esophageal squamous cell carcinoma, immunohistochemistry

## Abstract

Insulin-like growth factor-II mRNA-binding protein-3 (IMP3) is an important factor in carcinogenesis, although its clinical significance in esophageal squamous cell carcinoma (ESCC) remains unknown. The present study investigated the associations between IMP3 expression and the clinicopathological parameters. IMP3 expression was assessed in 191 resected ESCC specimens, and the associations between IMP3 expression in ESCC, the clinicopathological parameters and patient prognosis were examined. Using immunohistochemistry, 113 (59.2%) tumors were identified as IMP3-positive. IMP3 positivity correlated significantly with high pathological (p)Stage, pT stage and pN stage. The IMP3-positive patients exhibited a poorer prognosis compared with the IMP3-negative patients. In univariate analyses, histology [hazard ratio (HR), 1.94; 95% confidence interval (CI), 1.18–3.49; P=0.0082], pT (HR, 2.34; 95% CI, 1.55–3.62; P<0.0001), pN (HR, 2.85; 95% CI, 1.81–4.69; P<0.0001), lymphatic invasion (HR, 2.08; 95% CI, 1.26–3.70; P=0.0036), venous invasion (HR, 1.79; 95% CI, 1.21–2.64; P=0.0039), neoadjuvant chemotherapy (NAC) (HR, 2.01; 95% CI, 1.35–3.00; P=0.0005) and IMP3 expression (HR, 2.12; 95% CI, 1.40–3.29; P=0.0003) were significantly associated with overall survival. Using multivariate analyses, histology (HR, 1.87; 95% CI, 1.13–3.29; P=0.014), pN (HR, 2.19; 95% CI, 1.36–3.66; P=0.0010), NAC (HR, 1.88; 95% CI, 1.24–2.86; P=0.0028) and IMP3 expression (HR, 1.84; 95% CI, 1.18–2.93; P=0.0064) were significant prognostic factors. IMP3 may therefore be a prognostic factor for patients with ESCC who have undergone a curative resection.

## Introduction

In East Asian countries, esophageal squamous cell carcinoma (ESCC) is the major histological form of esophageal cancer. The disease is also one of the most lethal digestive tract malignances (1). In the majority of cases, the initial diagnosis of ESCC is made when the malignancy has already progressed to an advanced stage (1). Despite recent improvements in multi-treatment approaches, including surgery, radiotherapy and chemotherapy, the prognosis for patients with ESCC remains unsatisfactory (2). Predicting a prognosis by examining the clinicopathological characteristics remains difficult, even when using the tumor-node-metastasis staging system. This is due to considerable tumor variability and heterogeneity within the same pathological stage.

The IMP3 gene, also known as the K homology domain-containing gene (KOC) or L523S, encodes the IMP3 protein (3). IMP3 is located on chromosome 7p11.5 and encodes a 4350-bp mRNA and a 580-aa protein. IMP3 is expressed in the developing epithelium, muscle and placenta during the early stages of human and mouse embryogenesis, and low or undetectable levels of IMP3 are present in adult tissues (4,5). IMP3 has been shown to be overexpressed in testicular cancer, renal cell carcinoma, ovarian carcinoma, gastric cancer, colon cancer and adenocarcinoma of the lung (6–15). The IMP3 protein, together with IMP-1 and IMP-2, has different functions in various post-transcriptional processes, including mRNA localization, mRNA turnover and translational control (16–19). The IMP3 gene has previously been used as a marker to detect malignant cells in fine-needle aspirates (20). Additionally, in K562 leukemia cells, the inhibition of IMP3 has been shown to result in apoptosis, indicating that it may be vital for cancer cell survival (18). IMP3 is a prognostic biomarker in patients with endometrial serous carcinoma and renal cell carcinoma. In such cases, IMP3 expression appears to predict an increased likelihood of metastasis following surgery and a shorter metastasis-free survival time (8–11,15). However, to the best of our knowledge, the clinicopathological and prognostic significance of IMP3 expression in ESCC remains unknown. In the present study, the prevalence and clinicopathological significance of IMP3 expression were investigated with regard to overall survival (OS) and recurrence-free survival (RFS) in 191 patients.

## Materials and methods

### Patients and treatments

The present study examined 191 patients with pathologically confirmed primary ESCC who underwent surgical resection at the Osaka University Hospital (Osaka, Japan) between 1998 and 2007 (Table I). Approval for the study was obtained from the Ethics Committee of Osaka University Hospital. The study population consisted of 24 female and 167 male patients who ranged between 29 and 85 years of age (median, 62.7 years). All patients underwent a subtotal esophagectomy via a right thoracotomy, with a two- or three-field lymphadenectomy, with curative resection. None of the patients succumbed to post-operative complications. Of the 104 patients with lymph node metastases at the initial diagnosis, 86 received neoadjuvant chemotherapy (NAC), which consisted of two courses of 5-fluorouracil (700 mg/m^2^ on days one to seven), cisplatin (70 mg/m^2^ on day one) and Adriamycin (35 mg/m^2^ on day one). Following surgery, the patients were followed up every 3 months by physical examination and an analysis of serum tumor markers (squamous cell carcinoma antigen and carcinoembryonic antigen), every 6 months by computed tomography scanning and abdominal ultrasonography, and every year by endoscopy until tumor recurrence became evident. Patients exhibiting tumor recurrence received chemotherapy or chemoradiotherapy for as long as this regimen was systemically tolerated. The mean OS time was 41 months, and the mean RFS time was 39 months.

### Immunohistochemical analysis

IMP3 expression was examined in formalin-fixed, paraffin-embedded ESCC tissue sections by immunohistochemistry (IHC). One representative slide with the deepest tumor invasion was selected from each patient and examined by IHC. The tissue sections were deparaffinized in xylene and then rehydrated through a graded ethanol series. For antigen retrieval, the slides were incubated by autoclaving at 110°C in 10 mm Tris and 1 mm EDTA buffer (pH 9.0) for 20 min. Endogenous peroxidase activity was blocked with 0.3% H_2_O_2_ in methanol for 20 min and non-specific binding was blocked with 10% normal serum for 20 min. Subsequently, the tissue slides were incubated overnight with anti-IMP3 antibody (monoclonal mouse anti-human L523S; dilution, 1:200; Dako Cytomation, Carpinteria, CA, USA) at 4°C in a humidified chamber. The bound antibody was visualized using the Avidin/Biotin Complex Peroxidase Detection System (Vector Laboratories, Burlingame, CA, USA). Finally, the sections were incubated in 3,3′-diaminobenzidine tetrahydrochloride with 0.05% H_2_O_2_ for 3 min and counterstained with 0.1% hematoxylin. IMP3 staining for each ESCC sample was defined as positive when >10% of the cancer cells in the section were immunoreactive with the anti-IMP3 antibody. Staining was defined as negative when ≤10% of the cancer cells in the section were positive.

### Statistical analysis

Statistical analysis was performed using JMP software (JMP version 9.0.2; SAS Institute, Cary, NC, USA). The association between IMP3 expression and the clinicopathological parameters was assessed using the χ^2^ test. The RFS and OS were assessed using the Kaplan-Meier method and compared using the log-rank test. All the parameters that were found to be significant in a univariate analysis using the Cox proportional hazard model were entered into a multivariate survival analysis. P-values were derived from two-tailed testing and P<0.05 was considered to indicate a statistically significant difference.

## Results

### IMP3 expression in ESCC

**Figure 1:**
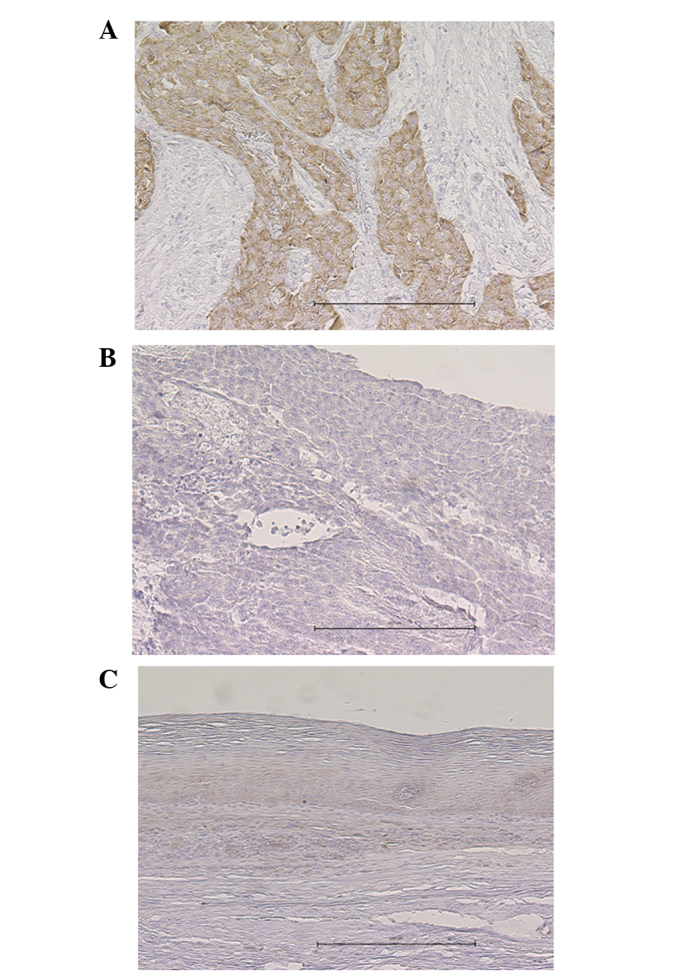
Representative images of IMP-3 expression, as determined by immunohistochemical staining. (A) IMP-3-positive esophageal squamous cell carcinoma exhibiting staining mainly in the cytoplasm of the tumor cells. (B) IMP-3-negative esophageal squamous cell carcinoma exhibiting almost no staining of the tumor cells. (C) Normal squamous epithelium negative for IMP-3. The black scale bar represents 250 μM. IMP3, insulin-like growth factor-II mRNA-binding protein-3.

A total of 191 samples that contained cancerous and non-cancerous lesions were evaluated for IMP3 expression using IHC. Of these, 113 (59.2%) showed positive IMP3 expression that was predominantly localized to the cytoplasm of the tumor cells, along with faint nuclear staining (Fig. 1A). The remaining 78 (40.8%) were negative for IMP3 expression (Fig. 1B). The positive staining was almost homogeneous in individual cancer foci and in different areas, such as in the surface, central and deepest areas, of the cancer lesions. By contrast, none of the normal squamous epithelia exhibited substantial IMP3 staining, although certain basal cells showed faint nuclear staining (Fig. 1C).

### Association between IMP3 expression and clinicopathological parameters

Table II lists the associations between IMP3 expression and the clinicopathological parameters. The IMP3-positive tumors were significantly associated with deeper tumor invasion and lymph node metastases compared with the IMP3-negative tumors (P=0.0001 and P=0.026, respectively). No significant associations were observed between IMP3 expression and other parameters, including age, gender, histology and use of NAC.

### Association between IMP3 expression and survival

**Figure 2:**
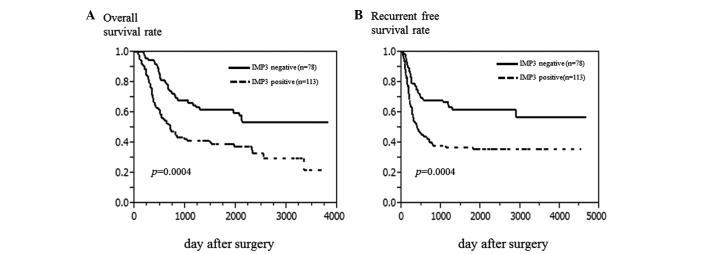
Survival curves according to IMP-3 expression. (A) Overall survival of all patients was plotted using the Kaplan-Meier method. (B) Recurrence-free survival of all patients. IMP3, insulin-like growth factor-II mRNA-binding protein-3.

The 5-year OS rate of the population was 48.5%. Patients with IMP3-positive tumors experienced a poorer 5-year OS rate compared with those with IMP3-negative tumors (39.3 vs. 61.7%, P=0.0004; Fig. 2A). Similarly, patients with IMP3-positive tumors experienced a poorer RFS rate compared with those with IMP3-negative tumors (35.7 vs. 61.9%, P=0.0004; Fig. 2B). By univariate analyses, histology [hazard ratio (HR), 1.94; 95% confidence interval (CI), 1.18–3.49; P=0.0082], pathological T stage (pT; HR, 2.34; 95% CI, 1.55–3.62; P<0.0001), pathological N stage (pN; HR, 2.85; 95% CI, 1.81–4.69; P<0.0001), lymphatic invasion (HR, 2.08; 95% CI, 1.26–3.70; P=0.0036), venous invasion (HR, 1.79; 95% CI, 1.21–2.64; P=0.0039), NAC (HR, 2.01; 95% CI, 1.35–3.00; P=0.0005), and IMP expression (HR, 2.12; 95% CI, 1.40–3.29; P=0.0003) were significantly correlated with OS (Table III). The seven parameters that demonstrated statistical significance (P<0.05) by univariate analysis were further analyzed by multivariate analysis. Multivariate analysis showed that pathological lymph node metastasis was the poorest prognostic factor (HR, 2.19; 95% CI, 1.36–3.66; P=0.0010), followed by NAC (HR, 1.88; 95% CI, 1.24–2.86; P=0.0028), histology (HR, 1.87; 95% CI, 1.13–3.49; P=0.014), and IMP3 expression (HR, 1.84; 95% CI, 1.18–2.93; P=0.0064) (Table III).

## Discussion

IMP3 is an RNA-binding protein and a KH domain-containing member of the IMP family. In mice, IMPs are primarily expressed during early embryogenesis and at mid-gestation, but they are not expressed in the majority of adult human tissues (3,4,22). IMP3 has been reported to function by regulating tumor cell proliferation, migration and metastasis. IMP3 has been shown to promote tumor cell proliferation through the upregulation of IGF2, a potent mitogenic factor previously shown to exert effects in a number of diseases (18,23,24). Studies have additionally found that IMP3 can exert a marked effect on cellular adhesion and invasion during normal development and during the development of cancers (25). For these reasons, strong IMP3 expression is regarded as an indicator of a poor prognosis (6,9,10,26,27). However, to the best of our knowledge, the clinicopathological and prognostic significance of IMP3 expression in ESCC has not been reported.

The present study demonstrated the positive immunoreactivity to IMP3 of 59.2% of ESCC surgical samples. Positive IMP3 expression was significantly associated with pathological factors associated with tumor progression [pT, pN and pathological stage (pStage)]. IMP3 was identified as a prognostic factor for OS. Although pT is generally considered to be an independent prognostic factor, this was not the case in the present series. In the present study, patients with advanced ESCC received NAC. Hence, the effect of pT was canceled by the effect of NAC in the multivariate analysis. This result was similar to that reported in other cancers (6,9–11,26,27). However, the clinical association between IMP3 and a worse prognosis of ESCC remains poorly defined. Yoshino *et al* (28) reported that IMP3 mRNA expression was associated with resistance to radiation therapy in ESCC cell lines. Further studies to investigate this should therefore be performed in the future.

Several characteristics of IMP3 indicate that it may be a potentially attractive prognostic marker. First, IMP3 IHC staining is a simple and reliable assay to perform (9). In the majority of cases, carcinomas are treated surgically, allowing chemotherapy and radiation therapy to be combined. Tumor tissues are thus routinely available for IHC staining using the monoclonal L523 antibody. The present study found that IMP3 IHC was reproducible and could be readily performed on ESCC tissues. The simplicity of this assay will enable a pre-operative diagnosis from the analysis of biopsy tissue. Regarding the polymerase chain reaction (PCR)-based method, IMP3 has been used as a molecular marker to predict peritoneal recurrence following curative surgery for gastric cancer (11), and PCR amplification of IMP3 from biliary structure specimens have been useful to distinguish between benign and malignant lesions (29). Furthermore, IMP3 has been considered a potential target for immunotherapy. A phase II study using a peptide vaccine therapy, which included IMP3, has been performed for patients with advance ESCC who failed to respond to standard therapies (30). It has been reported that the immune response induced by the vaccination may improve the prognosis for patients with advanced ESCC.

In conclusion, in the present study, IMP3, a novel mRNA-binding protein, was shown to be frequently expressed in ESCC. IMP3 expression was more commonly observed in ESCC patients with poor prognostic factors. IMP3 may be a potential IHC biomarker that can be used to evaluate the tumor progression and prognosis of ESCC.

## Figures and Tables

**Table I:** Characteristics of patients with ESCC.

Parameters	Value
Median age, years (range)	62.7 (29–85)
Gender, n (%)	
Male	167 (87.4)
Female	24 (12.6)
Histology of SCC, n (%)	
Poorly-differentiated	45 (23.6)
Moderately-differentiated	99 (51.8)
Well-differentiated	47 (24.6)
Pathological classification^a^, n (%)	
pT	
0	0 (0.0)
1	51 (26.7)
2	30 (15.7)
3	93 (48.7)
4	17 (8.9)
pN	
N0	68 (35.6)
N1	53 (27.7)
N2	35 (18.3)
N3	35 (18.3)
pStage	
0	0 (0.0)
I	39 (20.4)
II	53 (27.7)
III	63 (33.0)
IV	36 (18.8)

a According to the Union for International Cancer Control, 7th edition (21).

ESCC; esophageal squamous cell carcinoma; pN; pathological N stage; pT, pathological T stage; pStage, pathological stage.

**Table II:** Correlation between IMP3 expression and clinicopathological parameters.

	IMP3 expression, n (%)		
		
Parameters	Positive	Negative	P-value
Age, years			
<65	64 (33.5)	47 (24.6)	0.6179
≥65	49 (25.7)	31 (16.2)	
Gender			
Male	97 (50.8)	70 (36.6)	0.4191
Female	16 (8.4)	8 (4.2)	
Histology^a^			
Poor/moderate	89 (46.6)	55 (28.8)	0.1955
Well	24 (12.6)	23 (12.0)	
Neoadjuvant chemotherapy			
Yes	48 (25.1)	38 (19.9)	0.4654
No	65 (34.0)	40 (20.9)	
Depth of tumor invasion^b^			
pT1–2	35 (18.3)	46 (24.1)	0.0010
pT3–4	78 (40.8)	32 (16.8)	
Lymph node metastasis^b^			
pN0	33 (17.3)	35 (18.3)	0.0267
pN1–3	80 (41.9)	43 (22.5)	
pStage^b^			
I, II	67 (35.1)	46 (24.1)	0.0003
III, IV	46 (24.1)	32 (16.8)	

a Well-, moderately- and poorly-differentiated squamous cell carcinoma.

b According to the Union for International Cancer Control, 7th edition (21).

pN; pathological N stage; pT, pathological T stage; pStage, pathological stage; IMP3, insulin-like growth factor-II mRNA-binding protein-3.

**Table III:** Univariate and multivariate analysis of OS using Cox’s proportional hazard model.

		Univariate		Multivariate	
			
Parameter	Number of cases	HR (95% CI)	P-value	HR (95% CI)	P-value
Age (>65 years)	78/113	1.24 (0.84–1.84)	0.2766		
Gender (female/male)	24/167	1.05 (0.56–1.82)	0.8591		
Histology (poor-moderate/well)^a^	144/47	1.94 (1.18–3.49)	0.0082	1.87 (1.13–3.29)	0.0134
pT (T3,4/T1,2)^b^	110/81	2.34 (1.55–3.62)	<0.0001	1.28 (0.79–2.10)	0.3303
pN (N1–3, N0)^b^	123/68	2.85 (1.81–4.69)	<0.0001	2.19 (1.36–3.66)	0.0010
Lympathic invasion (present/absent)	148/43	2.08 (1.26–3.70)	0.0036	1.11 (0.62–2.08)	0.7354
Venous invasion (present/absent)	79/112	1.79 (1.21–2.64)	0.0039	1.22 (0.79–1.91)	0.3740
NAC (yes/no)	86/105	2.01 (1.35–3.00)	0.0005	1.88 (1.24–2.86)	0.0028
IMP3 expression (positive/negative)	113/78	2.12 (1.40–3.29)	0.0003	1.84 (1.18–2.93)	0.0064

a Well-, moderately- and poorly-differentiated squamous cell carcinoma.

b According to the Union for International Cancer Control, 7th edition (21).

OS, overall survival; pN; pathological N stage; pT, pathological T stage; HR, hazard ratio; CI, confidence interval; IMP3, insulin-like growth factor-II mRNA-binding protein-3; NAC, neoadjuvant chemotherapy.
